# Associations between Peripheral Thromboembolic Vascular Disease and Androgen Deprivation Therapy in Asian Prostate Cancer Patients

**DOI:** 10.1038/s41598-019-50522-4

**Published:** 2019-10-02

**Authors:** Yu-Chuan Lu, Chao-Yuan Huang, Huei-Ming Yeh, Jian-Hua Hong, Chao-Hsiang Chang, Chih-Hsin Muo, Shiu-Dong Chung, Teng-Kai Yang, Fu-Shan Jaw, Chi-Jung Chung

**Affiliations:** 1 Institute of Biomedical Engineering, National Taiwan University, Taipei, Taiwan; 2 Department of Urology, National Taiwan University Hospital, Taipei, Taiwan; 3 Department of Anesthesiology, National Taiwan University Hospital, Taipei, Taiwan; 4 Department of Urology, China Medical University and Hospital, Taichung, Taiwan; 5 Department of Medicine, College of Medicine, China Medical University and Hospital, Taichung, Taiwan; 6 Management Office for Health Data, China Medical University and Hospital, Taichung, Taiwan; 7 Division of Urology, Department of Surgery, Far Eastern Memorial Hospital, New Taipei City, Taiwan; 8 Graduate Program in Biomedical Informatics, College of Informatics, Yuan-Ze University, Chung-Li, Taiwan; 9 Surgery Department, Yonghe Cardinal Hospital, New Taipei City, Taiwan; 10 Department of Public Health, China Medical University, Taichung, Taiwan; 11 Department of Medical Research, China Medical University Hospital, Taichung, Taiwan

**Keywords:** Cancer, Prostate

## Abstract

This study aimed to investigate the risks of thromboembolic vascular disease following androgen deprivation therapy (ADT) administered to prostate cancer (PCa) patients. A total of 24,464 men with newly diagnosed PCa during 2000–2008 were recruited through a longitudinal health insurance database in Taiwan. All PCa patients were stratified into two: ADT and non-ADT groups. Patients with ADT treatment were grouped into three: surgical castration, chemical castration, and anti-androgen alone. The risks of pulmonary embolism (PE), peripheral arterial occlusion disease (PAOD), and deep vein thrombosis (DVT) were assessed in multiple Cox proportional-hazards regression with time-dependent covariates. During the 12-year follow-up period, incidence rates per 1000 person-years in ADT and non-ADT groups were 2.87 and 1.62 for DVT, 1.00 and 0.52 for PE, and 1.03 and 0.70 for PAOD, respectively. The DVT and PE risks were significantly increased in patients receiving combined androgen blockade (CAB) compared with the counterpart ADT non-recipients. After adjusting for potential risk factors, PCa patients receiving CAB had the highest PE risk (HR = 3.11), followed by DVT risk (HR = 2.53). The DVT risk remained elevated throughout the entire duration of chemical castration. However, high PE risk was observed in patients with ≤720-day treatment duration. No association was found between ADT and PAOD risks. Overall, the risks of PE and DVT were considerably heightened in Asian men subjected to CAB for PCa, whereas PAOD risk was unrelated to such treatments.

## Introduction

Androgen deprivation therapy (ADT) is typically used to treat metastatic and advanced prostate cancer (PCa). Numerous studies have demonstrated a heightened risk of various illnesses, such as diabetes mellitus^1^, cardiovascular disease^2^, cerebrovascular disease^3^, or myocardial infarction, in conjunction with ADT. The association between ADT and peripheral vascular disease (PVD) has also been investigated in the Western population, especially for peripheral arterial occlusive disease (PAOD), deep venous thrombosis (DVT), and pulmonary embolism (PE)^4–7^.

Various studies have also linked low testosterone levels in men to reduce fibrinolytic activity^8,9^. This finding suggests that ADT confers a hypercoagulable state and thus increases the risk of venous thromboembolism (VTE). Cancer itself is a risk factor for thromboembolic phenomena, responsible for an estimated 300,000 deaths annually^10^. Various oncologic treatments, such as surgery and chemotherapy, which directly prolong survival times, also increase the risk of VTE^11,12^.

The risk of PVD in Asians has been considered to be lower than that in Whites^13–15^. However, the impact of ADT on the PVD incidence among Asian PCa patients remains unknown. Given the lack of existing evidence, a population-based retrospective cohort study is conducted to assess the impact of ADT therapy on subsequent DVT, PAOD, and PE in Asian patients with PCa.

## Result

A total of 11,158 PCa patients, including 7903 (70.8%) and 3255 (29.2%) patients with and without ADT treatments, respectively, were analyzed in this retrospective cohort study. The ADT group appeared to be more advanced in age (73.2 ± 8.31 vs. 70.7 ± 9.84 years old) yet less likely to reside in the urban area (56.2% vs. 59.4%) compared with the non-ADT group (Table 1). For comorbidity, the ADT group with frequent hypertension history (50.0% vs. 47.7%) had better chances to avoid cancers than the non-ADT group (5.69% vs. 6.76%). Many patients in the ADT group also received chemotherapy and radiotherapy. By contrast, certain patients in the non-ADT group underwent radical prostatectomy. In the ADT group, most patients received chemical castration treatment (53.3%), especially CAB treatment, followed by oral anti-androgen treatment alone (43.9%) and surgical castration treatment (2.82%).

**Table 1:** Age and comorbidity distributions in PCa patients with ADT group and non-ADT group.

	ADT group N = 7903 (70.8%)		non-ADT group N = 3255 (29.2%)		*P*-value
Mean age, yr (SD)	73.2	(8.31)	70.7	(9.84)	<0.0001
Urbanization, n (%)					<0.0001
1 (highest)	2259	(28.6)	1060	(32.6)	
2	2184	(27.6)	871	(26.8)	
3	1151	(14.6)	509	(15.6)	
4	1305	(16.5)	445	(13.7)	
5 (lowest)	1004	(12.7)	370	(11.4)	
Comorbidity, n (%)					
COPD	2234	28.3	882	27.1	0.21
Other cancers	450	5.69	220	6.76	0.03
Heart failure	266	3.37	97	2.98	0.30
Hypertension	3951	50.0	1554	47.7	0.03
Nephrotic syndrome	1254	15.9	516	15.9	0.98
Stroke	865	11.0	351	10.8	0.80
Lower leg fracture or surgery	379	4.80	144	4.42	0.40
Certain medications, n (%)	32	0.40	20	0.61	0.14
Received chemotherapy	1243	15.7	259	7.96	<0.0001
Received prostatectomy	922	11.7	1319	40.5	<0.0001
Received radiotherapy	3439	43.5	859	26.4	<0.0001
ADT, n (%)					
Surgical castration	223	(2.82)			
Anti-androgen	3472	(43.9)			
Chemical castration					
GnRH agonist alone	1159	(14.7)			
CAB	3049	(38.6)			

Values expressed as n (%).

Certain medications include tamoxifen, thalidomide and erythropoietin.

CAB: combined androgen blockade.

### Deep vein thrombosis (DVT)

**Figure 1:**
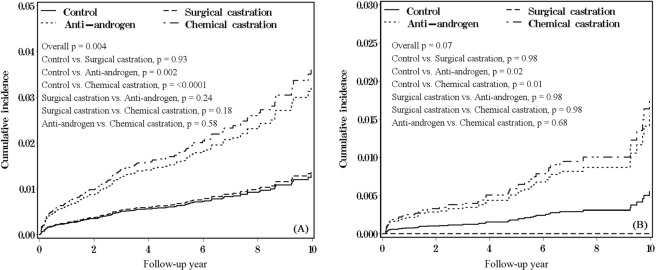
Cumulative incidences in different ADT treatments for (**A**) PE and (**B**) DVT.

During the study period, 92 and 37 patients with DVT development corresponded to incidence rates of 2.87 and 1.62 per 1000 person-years in the ADT and non-ADT groups, respectively (Table 2). The cumulative incidence in patients with anti-androgen and chemical castration treatments was significantly higher than that of the non-ADT group (p = 0.002 and <0.0001, respectively) (Fig. 1A). After adjusting for all variables in Table 1, the ADT group had 2.11-fold larger DVT risk (95% CI = 1.33–3.36) compared with that of the non-ADT group. The highest incidence of 3.47, 2.80, 1.64, and 1.60 per 1000 person-years for different ADT treatments was observed in patients with CAB, anti-androgen alone, surgical castration, and GnRH agonist alone treatments, respectively. Compared with the non-ADT group, patients with CAB treatment had the highest DVT risk (HR = 2.53; 95% CI = 1.45–4.42), followed by anti-androgen alone (HR = 2.17; 95% CI = 1.30–3.62), GnRH agonist alone (HR = 1.30; 95% CI = 0.45–3.77), and surgical castration treatment (HR = 0.96; 95% CI = 0.23–4.02) in multiple Cox models with time-dependent covariates. Overall, patients with total castration had about two-fold risk of DVT (HR = 2.06; 95% CI = 1.23–3.46). For the different durations of chemical castration treatment, patients with ≤720 or >720-day treatment had similar significantly higher DVT risk than that of the non-ADT group (HR = 2.28 and 2.31; 95% CI = 1.24–4.19 and 1.17–4.53, respectively). In the follow-up stratified analysis, patients with anti-androgen treatment had a significantly higher DVT risk than that of the non-ADT group only at two-year follow-up (HR = 6.38; 95% CI = 1.28–31.7) (Table 3).

**Table 2:** Incidences and hazard ratios for risks of PE, PAOD, and DVT in recipients of various ADT treatment for prostate cancer.

	Event no	Person-years	Rate (per 1000 person-years)	Adjusted HR (95% CI)^a^
Outcome: DVT				
Control	37	22811	1.62	Ref.
ADT overall	92	32021	2.87	2.11 (1.33–3.36)**
Surgical castration	2	1219	1.64	0.96 (0.23–4.02)
Anti-androgen	51	18209	2.80	2.17 (1.30–3.62)**
Chemical castration	39	12592	3.10	2.27 (1.32–3.89)**
GnRH agonist alone	4	2504	1.60	1.30 (0.45–3.77)
CAB	35	10089	3.47	2.53 (1.45–4.42)**
Total castration	41	13811	2.97	2.06 (1.23–3.46)**
Duration of chemical castration				
≤180 days	5	1152	4.34	2.78 (1.00–7.72)*
181–720 days	17	5882	2.89	2.16 (1.12–4.17)*
>720 days	17	5558	3.06	2.29 (1.17–4.50)*
≤720 days	22	7034	3.13	2.28 (1.24–4.19)**
>720	17	5558	3.06	2.31 (1.17–4.53)*
Outcome: PE				
Control	12	22896	0.52	Ref.
ADT overall	32	32158	1.00	2.10 (0.94–4.72)
Surgical castration	0	1230	0.00	NA
Anti-androgen	18	18295	0.98	2.25 (0.91–5.60)
Chemical castration	14	12634	1.11	2.51 (0.97–6.46)
GnRH agonist alone	0	2515	0.00	NA
CAB	14	10119	1.38	3.11 (1.19–8.16)*
Total castration	14	13863	1.01	2.07 (0.85–5.05)
Duration of chemical castration				
≤180 days	1	1155	0.87	2.59 (0.30–22.2)
181–720 days	7	5902	1.19	3.27 (1.09–9.76)*
>720 days	6	5576	1.08	1.95 (0.61–6.22)
≤720 days	8	7058	1.13	3.16 (1.10–9.07)*
>720	6	5576	1.08	1.95 (0.61–6.19)
Outcome: PAOD				
Control	16	22887	0.70	Ref.
ADT overall	33	32103	1.03	1.01 (0.53–1.92)
Surgical castration	0	1230	0.00	NA
Anti-androgen	22	18267	1.20	1.17 (0.59–2.34)
Chemical castration	11	12606	0.87	0.84 (0.38–1.89)
GnRH agonist alone	0	2515	0.00	NA
CAB	11	10092	1.09	1.01 (0.45–2.30)
Total castration	11	13836	0.80	0.78 (0.35–1.75)
Duration of chemical castration				
≤180 days	1	1154	0.87	1.26 (0.15–10.3)
181–720 days	4	5894	0.68	0.71 (0.23–2.21)
>720 days	6	5558	1.08	0.91 (0.34–2.48)
≤720 days	5	7049	0.71	0.78 (0.27–2.21)
>720	6	5558	1.08	0.92 (0.34–2.49)

*p < 0.05; **p < 0.01.

^a^Adjusted for all variables listed in Table 1.

**Table 3:** Crude and adjusted HR for outcomes among Anti-androgen alone, and CAB.

Variable	Control	Anti-androgen	Crude HR (95% CI)	Adjusted HR (95% CI)^a^	CAB	Crude HR (95% CI)	Adjusted HR (95% CI)^a^
	Rate, per 1000 person-years	Rate, per 1000 person-years			Rate, per 1000 person-years		
DVT							
1 year	3.42	2.69	1.35 (0.47–3.87)	1.17 (0.39–3.49)	2.76	1.49 (0.39–5.72)	1.27 (0.31–5.13)
2 year	0.55	3.50	6.46 (1.43–29.2)*	6.38 (1.28–31.7)*	3.32	6.13 (1.24–30.4)*	5.25 (0.94–29.5)
3 year	1.56	3.39	2.17 (0.74–6.34)	1.45 (0.48–4.36)	6.45	4.12 (1.43–11.9)**	2.02 (0.67–6.09)
4 year	1.12	0.39	0.35 (0.04–3.33)	0.26 (0.03–2.63)	2.73	2.44 (0.55–10.9)	2.14 (0.43–10.7)
5 year	1.43	1.44	1.01 (0.20–5.00)	0.90 (0.14–5.64)	0.00	NA	NA
PE							
1 year	0.98	1.79	6.00 (0.81–44.2)	4.73 (0.60–37.0)	0.00	NA	NA
2 year	0.00	0.64	NA	NA	1.66	NA	NA
3 year	0.31	0.00	NA	NA	1.17	3.78 (0.34–41.7)	4.04 (0.19–87.2)
4 year	0.37	1.16	3.09 (0.32–29.7)	1.53 (0.16–14.9)	1.36	3.63 (0.33–40.0)	1.72 (0.14–20.6)
5 year	0.95	0.00	NA	NA	0.86	0.90 (0.08–9.95)	1.63 (0.11–24.3)

*p < 0.05; **p < 0.01.

^a^Adjusted for all variables listed in Table 1.

### Pulmonary embolism (PE)

Approximately 32 and 12 patients with PE development in comprised the ADT and non-ADT groups, respectively, during the study period. Cumulative incidence in patients with anti-androgen and chemical castration treatments was significantly higher than that of the non-ADT group (p = 0.02 and 0.01, respectively) (Fig. 1B). The ADT group had 2.10-fold larger PE risk in the multivariable Cox model with time-dependent covariate (95% CI = 0.94–4.72) compared with that of the non-ADT group (Table 2). The highest incidence for different ADT treatments was observed in patients with CAB treatment (1.38 per 1000 person-years), followed by anti-androgen treatment (0.98 per 1000 person-years). Compared with the non-ADT group, ADT group patients with CAB treatment had the highest PE risk, followed by anti-androgen treatment (HR = 3.11 and 2.25; 95% CI = 1.19–8.16 and 0.91–5.60, respectively). Regarding the different durations of chemical castration treatment, patients with ≤720 or >720-day treatment had higher PE risk than those of the non-ADT group. However, only those with chemical castration ≤720 days had a significant statistical difference (HR = 3.16, 95% CI = 1.10–9.07).

### Peripheral arterial occlusion disease (PAOD)

A total of 33 and 16 patients with PAOD development comprised the ADT and non-ADT groups, respectively, during the study period. Such values correspond to incidence rates of 1.03 and 0.70 per 1000 person-years (Table 2). The ADT group had 1.01-fold larger PAOD risk in the multivariable Cox model with time-dependent covariate (95% CI = 0.53–1.92) compared with that of the non-ADT group. The highest incidence for different ADT treatments was observed in patients with anti-androgen alone treatment (1.20 per 1000 person-years), followed by CAB treatment (1.09 per 1000 person-years). ADT group patients with CAB or anti-androgen treatment had higher PAOD risk compared with that of the non-ADT group; however, this finding did not achieve statistical significance. For the different durations of chemical castration treatment, patients in the ADT group with ≤720 or >720-day treatment had a similar PAOD risk as that of the non-ADT group.

## Discussion

Asians have been considered to exhibit a low incidence of thromboembolic vascular disease^13–15^. However, the actual incidence for DVT, PE, and PAOD of Asian prostate cancer patients using ADT is unknown. In this large population-based study, our findings indicate that the DVT and PE risks were significantly increased in Asian PCa patients receiving anti-androgen alone and CAB compared with those of the counterpart ADT non-recipients. In the multiple Cox model with time-dependent covariate, patients receiving CAB had higher risk of PE (HR = 3.11) and DVT (HR = 2.53) respectively compared with that of other types of ADT. The risk of DVT remained elevated throughout the entire duration of chemical castration. However, a high PE risk was observed in patients with a treatment duration of ≤720 days. The systematic review of the relevant research indicated that the present study is the first to examine the absolute and relative risks of DVT, PE, and PAOD in prostate cancer patients receiving ADT by using an East Asian population database.

In another population-based study drawn from the Swedish National Prostate Cancer Registry, patients with advanced PCa receiving ADT showed an increased risk of VTE, which is in contrast to a matched male comparator group without PCa^5^. However, no discernable relation existed between ADT and arterial embolism. Although ADT and heightened risk of PAOD were previously linked in patients with non-metastatic PCa, these outcomes were consistent with the present findings^4^. A recent publication reported that the use of GnRH agonists was associated with 52% increased risks of VTE, whereas bilateral orchiectomy showed no association^6^. GnRH agonists and oral antiandrogens contributed to higher risks of VTE (HR = 2.69) compared with only GnRH agonists (HR = 1.52) or oral antiandrogens (HR = 1.43). The present findings also demonstrated a high risk of incident PE and DVT with the use of CAB, which is consistent with the previous result.

The increased risk of DVT was most prominent at the second and third years of therapy, and only a few DVT events were observed after three years of chemical castration. Although high PE risk was only observed in patients with a treatment duration of ≤720 days, the DVT risk was significantly elevated in patients with ≤720 or >720-day CAB treatment than that of ADT non-users. Previous studies reported that long durations of ADT were associated with a large number of thromboembolic events and risk^7^. Hu *et al*. observed no cumulative risk for VTE associated with long ADT duration in non-metastatic prostate cancer^4^. Further research is needed to understand the mechanisms behind the observed associations.

The underpinnings of PE, DVT, or PAOD risk in this setting are partially understood. Thromboembolism is a common and multifactorial complication among cancer sufferers and dependent upon patient characteristics, type of malignancy, and nature of anti-cancer treatments rendered. Some investigations have implicated small procoagulant microparticles (MPs) as pathogenetic mediators, which circulate in the milieu of cancer coagulopathy and promote thrombus formation^16,17^. A recent study further confirmed the association between tissue factor-bearing MPs and clotting disorders in patients with PCa^18^. In limited testing, significant elevation of quantifiable MPs was documented in patients receiving ADT for PCa, providing additional support to this concept^19^. By contrast, testosterone was believed to manifest a cardioprotective effect^2,20,21^. Preliminary experimental findings suggested that androgens exert regulatory control of arterial thrombosis through their influence on platelet activation^22^, and high testosterone concentrations are associated with increased levels of antithrombin-3^23,24^. Thus, testosterone suppression may induce a hypercoagulable state, leading to cardiovascular disease^25^.

Notably, the deprivation of endogenous androgens conferred by various modes of therapy may be non-identical. Although orchiectomy and GnRH agonist reduce testosterone levels, several GnRHa-treated patients (15%) in one study registered levels beyond those of bilateral orchiectomy^26^. Such persistence of androgenic activity likely reflects adrenal production and breakthrough testicular release. Microsurges in testosterone levels have been known to occur in approximately 6% of GnRHa-treated patients^27^. CAB is suitable to maximally reduce testosterone level/function, whereas GnRHa alone may result in high testosterone levels. This plausible mechanism may explain the association of VTE with CAB instead of GnRH agonist alone or surgical castration.

The present study manifested several acknowledged limitations. Some clinical factors, such as Gleason score and tumor stage or grade, which were ignored in the Taiwan National Health Insurance Research Databases (TNHIRDs), were partially considered in the present analysis. Consequently, we could not compare the difference of the Gleason score and tumor stage between the ADT and non-ADT groups. The effect of disease burden/state on thromboembolic vascular disease cannot fully be evaluated. Furthermore, other potential biases cannot be excluded due to the retrospective nature of the present study, which may only approve association and not causality. The PE and PAOD incidence manifested in the present study subjects was low^28^. Accordingly, any selective investigation of GnRH agonist or anti-androgens and PE risk was prohibited. Small sampling sizes of targeted subsets may have contributed in this regard.

In this retrospective cohort analysis, CAB was associated with increased risks of subsequent PE and DVT in Asian patients with PCa. Nevertheless, surgical castration or GnRH agonist alone showed no association with subsequent PE and DVT. Any type of ADT showed no correlation with subsequent PAOD risk.

## Materials and Methods

### Data source

A Longitudinal Health Insurance Database for catastrophic illness patients (LHID-CIP) from the Taiwan National Health Insurance Research Databases was used in this retrospective cohort study. TNHIRDs were set up by the Taiwan National Health Insurance Administration Ministry of Health and Welfare (TNHIAM-HW) from a health insurance program, which included almost all residents in Taiwan. LHID-CIP included all medical claims and treatments from 1997 to 2011 for each catastrophic illness patient. Patients were defined as catastrophically ill ones by an approved specialist based on the guideline in TNHIAM-HW. Catastrophic illness included 30 diseases, such as malignancy, chronic renal failure with regular dialysis, and autoimmune disease. The definition of disease and medicine use in LHID-CIP is based on the International Classification of Diseases, Ninth Revision, Clinical Modification (ICD-9-CM) and the Anatomical Therapeutic Chemical Classification System (ATC code). LHID-CIP is a second-hand database because the identification of insurant was re-coded. All patient data were de-identified and encrypted before its release for research purposes. This study was also approved by the Research Ethics Committee of China Medical University Hospital (IRB CMUH104-REC2-115(CR-4)). All methods were performed following the relevant guidelines and regulations of the China Medical University and Hospital.

### Study participants

**Figure 2:**
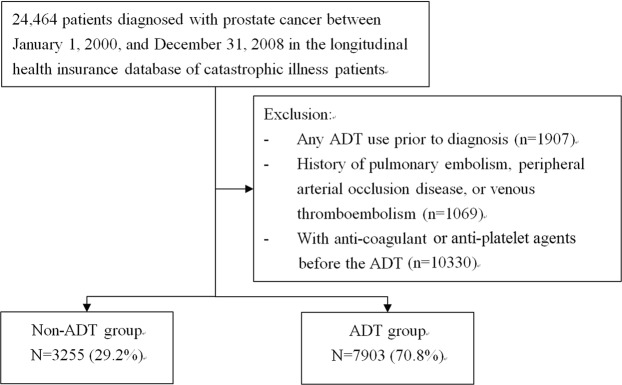
Study flow chart.

A total of 24,464 patients newly diagnosed with prostate cancer (ICD-9-CM 185) in 2000–2008 were collected from LHID-CIP, and the date for cancer diagnosis was defined as index date. Figure 2 presents the details of the study protocol for recruitment. Patients were further classified into ADT and non-ADT groups. Patients with ADT treatment before the index date, those with PE, PAOD, or VTE history before the index date, or those with anti-coagulant or anti-platelet agents before the ADT treatment were excluded.

### ADT treatment

ADT treatment included bilateral orchiectomy, gonadotropin-releasing hormone agonist (GnRH) treatment, and oral anti-androgen treatment. GnRH agonist contained leuplin (ATC code L02AE02), zoladex (ATC code L02AE03), decapeptyl (ATC code L02AE04), and diphereline (ATC code L02AE04). Oral anti-androgen contained cyproterone acetate (ATC code G03HA01), flutamide (ATC code L02BB01), and bicalutamide (ATC code L02BB03). Patients receiving medication more than three months were defined as users. Patients with ADT treatment were grouped into three: surgical castration, anti-androgen alone, and chemical castration. The chemical castration group included GnRH agonist alone and GnRH agonist CAB. Patients with GnRH agonist or bilateral orchiectomy combined oral anti-androgen treatment over three months were defined as CAB users.

### Risk factors and outcomes

In this study, the risk factors included age, urbanization, comorbidity, and usage of certain medications. Urbanization was classified into five levels based on the report of Lui: “Level 1” was the highest urbanization, and “Level 5” was the lowest urbanization^18^. Comorbidity included chronic obstructive pulmonary disease (ICD-9-CM 491, 492, and 496), other cancer (ICD-9-CM 140–184 and 186–208), heart failure (ICD-9-CM 428), hypertension (ICD-9-CM 401–405), nephrotic syndrome (ICD-9-CM 580–590), stroke (ICD-9-CM 430–438), and lower leg fracture or surgery (ICD-9-CM 820, 821, and 823; ICD-9-CM surgery codes 81.51–81.54). Certain medications contained tamoxifen (ATC code L02BA01), thalidomide (ATC code L04AX02), and erythropoietin (ATC code B03XA01). Clinical chemotherapy information, such as mitoxantrone, docetaxel or cabazitaxel, radical prostatectomy, and curative/palliative radiotherapy, was also considered in the final analysis.

The interesting outcomes included PE (ICD-9-CM 415.1), PAOD (ICD-9-CM 443.9), and DVT (ICD-9-CM 453.8). Patients were followed up from the index date until each outcome development. If patients had no outcome development, then they were followed up until the date for withdrawal from the health insurance program or the end of 2011.

### Statistical analysis

Number and percentage were presented for categorical variables between patients with and without ADT treatment. Chi-square test was used to examine the difference of categorical variables between patients with and without ADT treatment. Student’s t-test was also utilized to examine the average age difference between the two cohorts. Outcome incidence was the sum of outcome occurrence obtained by dividing the sum of the follow-up year (person-years). The ADT treatment condition may change with follow-up duration. Therefore, multiple Cox proportional-hazards regression with time-dependent covariate was used to assess the risks of various outcomes in all analyses and reduce immortal time bias. The cumulative incidence curve was plotted using the Kaplan–Meier method and tested through crude Cox proportional-hazards regression with time-dependent covariate. Subsequently, the effects of ADT treatment on risks of various thromboembolic vascular diseases and the stratified analysis of the duration of chemical castration use were adjusted for all variables listed in Table 1. These variables include age, urbanization, comorbidity, and receiving chemotherapy, prostatectomy, and radiotherapy in the multiple Cox proportional-hazards regression with time-dependent covariate. The DVT and PE risks in anti-androgen and CAB treatments stratified by follow-up years were also estimated. SAS software version 9.4 (SAS Institute, Cary, NC) was used in all statistical analyses. The significant level was set to p < 0.05 at two-tailed tests.
